# Generative AI, UK Copyright and Open Licences: considerations for UK HEI copyright advice services

**DOI:** 10.12688/f1000research.143131.1

**Published:** 2024-02-22

**Authors:** Andrew Johnson

**Affiliations:** 1 The University of Sheffield, Sheffield, England, UK

**Keywords:** copyright, artificial intelligence, AI, generative AI systems, open access, open licences

## Abstract

With the enormous growth in interest and use of generative artificial intelligence (AI) systems seen since the launch of ChatGPT in autumn 2022 have come questions both about the legal status of AI outputs, and of using protected works as training inputs. It is inevitable that UK higher education institution (HEI) library copyright advice services will see an increase in questions around use of works with AI as a result. Staff working in such library services are not lawyers or able to offer legal advice to their academic researchers. Nonetheless, they must look at the issues raised, consider how to advise in analogous situations of using copyright material, and offer opinion to researchers accordingly. While the legal questions remain to be answered definitively, copyright librarians can still offer advice on both open licences and use of copyright material under permitted exceptions. We look here at how library services can address questions on copyright and open licences for generative AI for researchers in UK HEIs.

## 1. Introduction

This article was prompted in part by an audience question at a recent library presentation, asking how open licences fit with artificial intelligence (AI). To answer the question of how library advice services in UK higher education institution (HEIs) can advise researchers to assess any copyright risks in their use of, or creation of their own, generative AI systems, we will look at the following research questions:

• Does the AI make copies of any copyright works used to train it?
• Does the AI create outputs that are copies of one or more copyright works used to train it?
• Does the AI further communicate any copies of copyright works online?
• Does the AI attribute the works used to train it?

To begin, we must define what we mean by open licences, copyright, and AI.

With regard to AI, we mean the text, image, music and video generative software that has seen a huge increase in attention of late. This includes products such as OpenAI’s ChatGPT and DALL-E2, Stable Diffusion, Google Bard, Adobe Firefly, and others. AI systems might be Large Language Models (LLMs, e.g., ChatGPT), or may rely on diffusion image generating technology (e.g., DALL-E, Stable Diffusion). We are specifically concerned with any generative AI software trained from a corpus of copyright protected works.

Turning to open licences, the presentation at which the AI query arose concerned Creative Commons (CC) licences, perhaps the most frequently encountered open licences for academic authors. They are the staple licences under which many of the research outputs from UK HEIs are published, due to the combination of Research Excellence Framework (REF) requirements and funder open access mandates (e.g., The Wellcome Trust ^1^ and UK Research and Innovation (UKRI) ^2^). The six main licences allow reuse under clearly defined terms, ranging from the most open Attribution (CC BY) to the most restrictive Attribution NonCommercial NoDerivatives (CC BY-NC-ND).

In addition to the above licences, we will consider works available under the CC0 Public Domain Dedication. CC0 waives all copyright and related rights in a work to the greatest extent permitted by law, and allows the work to be treated as public domain i.e., no restrictions on use or requirement to attribute ^3^ (though to avoid allegations of plagiarism, public domain works should still be cited in line with usual academic norms). There are also numerous open software licences, ^4^ the main principles of which apply in a similar way to those of the CC licences under discussion. Key questions for any open licence are:

• Does the licence permit:
  ○ Copying?
  ○ Distribution in any or all formats?
  ○ Communication online?
• Does the licence require attribution?
  ○ If yes, how must it (and/or the creator) be attributed?
• Does the licence allow commercial (i.e., profit-making) use?
• Does the licence allow derivatives (adaptations) of the licensed work to be made?
  ○ If yes, are there restrictions on how derivatives must be licensed and/or attributed?

In terms of copyright, we limit ourselves to the current situation as we see it in the UK. Copyright as an intellectual property (IP) right, and how the right may be infringed, is set out in the Copyright, Designs and Patents Act 1988 (CDPA) and the numerous amendments made since it came into force. The owner of copyright in a qualifying work has the right to prevent others doing any of the restricted acts – copying, publishing and distributing copies, playing, performing, renting or lending, adapting and communicating online. Set against these are certain permitted exceptions, under which others can use copyright works provided they adhere to the terms of the exception, do not prevent the rights owner exploiting their work in the usual manner, and do not compete economically with the original work.

We should also note the Copyright and Rights in Databases Regulations 1997. At s.6 this defines a database as ‘ *a collection of independent works, data or other materials which – (a) are arranged in a systematic or methodical way, and (b) are individually accessible by electronic or other means.’* The important points are that while individual copyright works might be included in a database, the whole collection may have protection under database right if there was, as defined at s.13(1) ‘ *a substantial investment in obtaining, verifying or presenting the contents of the database’.* If a person, without the database owner’s consent, ‘ *extracts or re-utilises all or a substantial part of the contents of the database*’, including by ‘ *the repeated and systematic extraction or re-utilisation of insubstantial parts of the contents*’, this infringes the database right. Furthermore, the database can, in theory, qualify as a copyright literary work if the selection and arrangement of the contents represents the author’s own intellectual creation.

Having established the terms and definitions to be used we will now address the four research questions and how the answers to these inform what advice should be given.

## 2. Does the AI make copies of any copyright works used to train it?

If an AI, or anyone, reproduces a copyright protected work beyond what is permitted under legal exception this can infringe the reproduction right. The exception to which we might turn when looking at AI training is s.29A CDPA, the exception for text and data mining (TDM). The conditions of that exception are that it is limited to non-commercial use, requires lawful access to the work, and any copies made cannot be transferred onward or be further used for any new purpose. Setting aside concerns TDM can facilitate academic data laundering ^5^ that bypasses non-commercial restrictions, we must look at another aspect of the AI training process which may affect what is permissible. That is whether the training involves multiple copying. For clarity, we should note we are not claiming all, or any specific, AI systems make such copies. Rather, we are considering what copyright issues researchers might conceivably encounter in using or creating generative AI systems, and how relevant library services should advise accordingly.

A corpus of online copyright works may be web-scraped and analysed for non-commercial scientific research. Any data generated may be shared openly and either have no copyright or be open access. If a commercial actor then uses that data, this in itself may not be creating further copies of the originally scraped works, so is not infringement of copyright. However, if the commercial actor makes any further copies of the works in training their AI, this is not covered by the TDM exception that allowed the initial research and is potentially infringing. For example, re-scraping or copying works to match this to metadata in a public domain database could prove infringing as it would not be covered by s.29A. So academic-commercial partnerships, or research activities making profit – directly or indirectly – from an AI system, cannot rely on the UK TDM exception to make copies as part of the system’s training. A suitable licence is required in such cases where copyright works form the training corpus.

In addition, the provisions of CDPA s.28A appear to offer little protection for copying of input training works. This permits transient or incidental copying as an essential part of a technical process, so may at first seem ideal for covering AI training. This exception only applies where all these conditions apply:

(it enables)…’ *a transmission of the work in a network between third parties by an intermediary; or … a lawful use of the work; and which has no independent economic significance.*’ ( CDPA s.28A)

This is intended to allow transient copying as must occur, for example, to allow the web to function. Where an AI system uses copyright material “in-house” as part of what is ultimately a for-profit business model this would not meet all the requirements above. This exception supports transient copying for non-commercial research use under s.29A, but only insofar as the reproduction is limited to that purpose - not for any further copying, training or communication.

So, what can be relied upon? Material licensed under the six main CC licences could be used for AI training. If the licence is a non-commercial one, then material could only be used if the AI system usage is not profit-making. However, problems of scale would be encountered if copies are shared, as the individual attribution requirements of CC licences might reasonably simply be met for a small number of works, but not for a large training corpus (see also Section 5 below). Avoiding onward distribution requiring attribution is important if using works available under Creative Commons licences. Anything available under CC0, or already in the public domain in the territory where the copying is taking place, can be freely copied and used to train – with one final caveat to consider.

That caveat is database right. A database may consist of uncopyrightable data, or copyrightable works, or a combination of both. While you might copy a database whose individual constituent works are open licensed or public domain without infringing copyright in the works themselves, you could still infringe database right. If the database represents a substantial investment on the part of the creator, then irrespective of the copyright status, or licence, of the individually searchable parts, repeated extraction of small parts can still infringe without the owner’s consent. A website collating a large body of images and associated metadata could qualify as a database. Repeatedly accessing and copying excerpts of the database – i.e., the web archive – could therefore be extraction or re-utilisation of a substantial part.

Databases can be openly licensed, much as their constituent parts can be, so if using a database for AI training it would be wise to choose one available under a permissive licence, or one available in a territory where no legal database right subsists – the UK and Europe have had database protection since 1996, ^6^ however many other territories do not.

## 3. Does the AI create outputs that are copies of one or more copyright works used to train it?

Here the waters are somewhat murkier. There are articles providing in-depth legal analysis (see for example Guadamuz, 2023 ^7^). Here we will limit our analysis to the more straightforward issues of UK copyright as we would expect a HEI copyright service to address them.

Due to the way AI systems generate outputs it is extremely rare for training inputs to be reproduced exactly (see for example Somepalli et al., 2022 ^8^ for diffusion image models, or Liang et al., 2022 ^9^ for LLM text regurgitation), however a work does not need to be reproduced in its entirety for there to be infringement. The CDPA only requires copying a substantial part (CDPA s.16(3a)) for infringement to occur. While some may believe Temple Island Collections Ltd v New English Teas Ltd & another [2012] EWPCC 1 ^10^ takes the protection of ideas or “style” too far, it stands as an example of how partial copying can be infringing in the UK. A user asking a generative AI to create an image in a particular artists’ style may not lead to an output that infringes any copyright, however if the prompt leads to close fitting to an existing work, or clearly reproduces a substantial part of an existing input too closely, this might infringe. It should be noted Temple Island is a very narrow decision, due to the judge basing his decision in part on the causal link, as he saw it, between the two works. ^11^ Whether a causal link would be found between an output substantially similar to a training input, and what effect – if any – the prompt used would have, remains to be determined.

Open licences are more helpful in the case of outputs. Reproduction of a substantial part of a work available under open licence is unlikely to be problematic and should be covered by the licence terms. Similarly, public domain or CC0 works may be freely reproduced. Care must be taken with some licences (e.g., non-commercial or share-alike). Any terms and conditions applied to the AI end-user’s reuse of the work their prompt generates should, if it substantially copies an input work, be compatible with the licence applying to that input. For example – and exceptionally unlikely this may be – if an output copies a substantial part of an input that is licensed non-commercially, that non-commercial licence might apply to the end-user’s reuse of the parts of their output that are a reproduction of the original.

All this immediately raises the objection that the AI is not copying as such, but rather only adding data in minimum amounts to create a new output, via an as-yet imperfectly understood process. ^12^ Building pixels into a representation of a dog chewing on a hat, in response to a prompt of ‘picture of a dog eating a hat’, that by chance matches closely or exactly to an original copyright work, can only infringe if that work was part of the training data, as the AI would have to have access to the allegedly infringed work to have copied it. However, if it is in the training data, then regardless of how the system generates outputs it may be difficult to argue against a challenge of copyright infringement if a substantial part of a trained work is reproduced.

Consequently, training on works that are public domain, openly licensed or under compatible reuse permissions must be the recommendation. This avoids any potential issues of infringing copying by commercial TDM in training inputs and negates any issues of infringement by outputs recreating a work in the training dataset too closely. This will, presumably, be the route chosen by Adobe with Firefly, for which they have offered their business users indemnity against copyright challenges. ^13^

From the observations above, we might conclude originality appears the important consideration in determining the potential for infringement – but possibly not so in determining copyright subsistence in outputs. We will briefly address the question of copyright ownership of outputs, and where this might subsist the further question of how such right is assigned and licensed.

The CDPA at s.9(3) allows for the possibility of copyright in computer-generated works in the UK, where such a work is, by virtue of the author, in theory qualifying for UK copyright. Computer generated is helpfully defined at CDPA s.178 as meaning a work with no human author. The copyright owner – author – is taken as being “ *the person by whom the arrangements necessary for the creation of the work are undertaken*”. ^14^ This seems ideally suited to allowing copyright in AI generated works. Duration of copyright in such works is fifty years from the end of the year the work was made. This shorter duration could reflect the reduced human skill and labour, or intellectual creation, required in the making of the work. It may also reflect that such literary, dramatic, musical or artistic works do not require the same originality to qualify for copyright. ^15^

More attention has been directed to analysing the question of whether AI generated works might qualify for copyright subsistence in the USA, ^16^ and how this should be registered, ^17^ than on the issue of who should own that right in the UK. For now, some commercial AI system owners seem confident that if outputs qualify for UK copyright then the system owner can choose how to licence such outputs. Copyright in prompts – so-called prompt engineering – is seemingly enjoying a moment of popularity despite some doubts about its future. ^18^ Court of Justice of the European Union (CJEU) precedent from Infopaq ^19^ allows even short combinations of words to qualify for copyright if they represent the author’s own intellectual creation. Despite this, ownership of any possible copyright in a prompt does not necessarily entail ownership of the output work. It is debatable whether the prompter contributed the correct intellectual creativity, or labour skill and judgment, to have made the arrangements necessary for the creation of the work, or whether they have in effect only “ *played the game*”. ^20^ Until greater clarity arises on this point, whether by guidance from the UK Intellectual Property Office, statutory amendment or judicial interpretation, this seems open to interpretation depending on how the system concerned is owned and operated.

Even if all input into the system’s training is public domain, new works created may qualify for copyright. The terms and conditions of any existing system researchers use should be noted carefully – for example, Midjourney ^21^ images created under the free trial terms are licensed CC BY-NC, with copyright owned by the company.

## 4. Does the AI further communicate any copies of copyright works online?

For outputs shared online by an end-user, who entered the prompt(s) to cause generation of the output, the licence terms applied to that output work will govern how they can use it. An output that (by virtue of s.9(3) and the qualifying status of the creator) has its own copyright and does not, in whole or substantial part, reproduce any input, can be communicated in accordance with the rights owner’s licence, and the terms and conditions of the AI system.

Further communication is unlikely to present a problem where the output is not a (substantial) copy of any copyright-restricted input, and both the owner of rights in the output (if any) and the terms of use of the system permit it. It is difficult to think of a situation where an AI might communicate inputs onward online other than as a substantially similar output. Communicating inputs directly would almost certainly infringe unless those inputs were public domain or (openly or otherwise compatibly) licensed.

## 5. Does the AI attribute the works used to train it?

We have already touched on the issue of attribution, so there is little to add beyond a brief look at outputs that recreate a substantial part of an input. As noted, this appears very unlikely, but theoretically possible. This raises the issue of how such a reproduction should attribute the original.

CC0 and the public domain are certainly the AI trainer’s friend once again, with no attribution required. What, though, of a work openly licensed under CC BY 4.0? Any onward sharing, or sharing of an adaptation, should include attribution. For AI systems to identify outputs that reproduce a substantial part of a protected input, and add suitable attribution at the point of generation, is surely impracticable. Breach of licence terms through failure to attribute is, at least in theory, possible. For version 4.0 CC licences this could be remedied by adding suitable attribution on demand from the licensor. Earlier CC licences could result in a permanent breach of the licence terms. Any possible risk from this is difficult to quantify, however the history of so-called copyright “trolls” exploiting ^22^ this seeming loophole in early CC licences is real and has led to many infringement actions.

## 6. Summary

The clear conclusion is that to be free of any concerns about copyright (or database right) infringement in the UK, researchers should use (or if training their own, create) an AI system trained entirely on public domain or suitably open-licensed works and databases. One possibility would be to use bespoke licensing covering use under predefined conditions. A body of works with clear licence terms allowing non-commercial educational use, with only blanket attribution of the corpus being required, would be a perfectly sensible option. A body of student coursework or dissertations, stored in a non-public intranet, could conceivably be such a corpus if the terms under which the students licence their IP to the institution as part of their enrolment are compatible.

Where either input works or the data source used for training is copyright protected, risk must creep in until the current situation is made clearer. The resolution of the existing legal challenges ^23^ may provide greater clarity.

## Data Availability

No data are associated with this article.
