# Supplementary material for: Generative AI, UK Copyright and Open Licences: considerations for UK HEI copyright advice services
Source: F1000Res. 2024 Feb 22;13:134. [Version 1] doi: 10.12688/f1000research.143131.1 (PMC11109589; doi:10.12688/f1000research.143131.1)
Supplement: Peer review history

# Reviewer report: Reviewer response for version 1

**DOI:** 10.5256/f1000research.156761.r274058

**Published:** 2024-05-10

**Authors:** Nicola Lucchi

**Affiliations:** 1 Universitat Pompeu Fabra, Barcelona, Catalonia, Spain

The article discusses the implications of using copyrighted materials in generative AI systems within UK Higher Education Institutions (HEIs). It provides a detailed analysis of the legal frameworks governing copyright in the UK, particularly as they apply to AI-generated content and the use of such content in academic settings. The article is structured around key legal questions that arise in the context of AI and copyright, including whether AI makes unauthorized copies of copyrighted works, whether AI outputs might themselves be copyright infringements, and the licensing implications of using copyrighted material to train AI.

 Although the article is rather short, it is well-researched and presents a thorough analysis of the copyright issues associated with the use of AI in academic environments. It effectively uses legal statutes and case law to discuss potential legal outcomes and the responsibilities of copyright advisors in HEIs. The originality of the article is evident in its specific focus on the role of HEI copyright advisors, providing nuanced insights into how these professionals can manage legal risks associated with AI technologies.

 The factual statements in the article are accurate and well-supported by citations. The author refers to relevant legal documents, case law, and current scholarly work to back up claims, providing an appropriate evidential base for the discussion.

 The conclusions drawn in the article are mostly balanced and justified based on the presented arguments. They provide practical, actionable advice for HEI copyright advisors based on a thorough analysis of the legal issues discussed. However, as noted by the other peer reviewer, the article could benefit from a broader discussion of non-transparent AI tools and the evolving legal landscape.

 Recommendations for Improvement: Given the rapid development in the field of AI and copyright, the article could be strengthened by referencing additional current literature. This would ensure that the article remains relevant and provides the most up-to-date advice possible.

 That said, the article is certainly a valuable contribution to the literature on copyright and AI, especially in the academic context, with some minor improvements.

Is the topic of the opinion article discussed accurately in the context of the current literature?

Yes

Are arguments sufficiently supported by evidence from the published literature?

Yes

Are all factual statements correct and adequately supported by citations?

Yes

Are the conclusions drawn balanced and justified on the basis of the presented arguments?

Yes

Reviewer Expertise:

Comparative law; Intellectual property law; Law & Technology; Media Law

I confirm that I have read this submission and believe that I have an appropriate level of expertise to confirm that it is of an acceptable scientific standard.

# Reviewer report: Reviewer response for version 1

**DOI:** 10.5256/f1000research.156761.r254872

**Published:** 2024-03-26

**Authors:** Jane Secker

**Affiliations:** 1 City University of London, London, England, UK

There is a huge amount of interest in copyright and AI - and copyright advisors in UK universities are getting a lot of questions - so this article is extremely timely and I suspect it's going to be of interest.

 It is not a legal analysis but it does point to case law and others who undertake legal analysis. It also looks in some detail at the text and data mining exception and whether this can be relied on by those who are using or creating Generative AI.

 The starting premise of the four questions that arose at a library event are all helpful. While I don't want to expand on the remit of the paper - I wonder if it needs to be clear that much of what it is discussing is relevant if an academic wants to build a GenAI - so encouraging them to rely on CC Licensed content or content in the public domain. I think it is worth acknowledging that the concerns extend beyond these questions, particularly when academics might be using a host of GenAI tools that are not transparent about the sources they are trained on - in light of cases such as the New York Times case - we could see some of these tools having to be more transparent.

 However there are also questions about whether you can protect works created by an AI with no human creator. And what to advise academics who might be tempted to feed their own research data into an AI to help analyse it (that is likely to contravene the ethical approval they have for a project aside from any copyright concerns.).

 While I don't want the author to widen the scope, it might be worth just flagging up there are increasing numbers of copyright issues that are arising and that copyright advisors are going to need to try and stay up to date with the case law and evolving landscape - which is a real challenge. I wonder if it might end with some practical - where to go to keep up to date. LIS-Copyseek (the Jiscmail list for copyright queries) remains very lively and many AI related issues are being discussed. There are of course various briefings and Jisc are keeping the sector up to date on AI. 

 But I welcome this publication, with just a few amendments to the introduction and conclusion just to flag up some of the wider issues and to point people to sources of advice.

Is the topic of the opinion article discussed accurately in the context of the current literature?

Yes

Are arguments sufficiently supported by evidence from the published literature?

Yes

Are all factual statements correct and adequately supported by citations?

Yes

Are the conclusions drawn balanced and justified on the basis of the presented arguments?

Partly

Reviewer Expertise:

Copyright and new technologies, information and digital literacy, copyright literacy

I confirm that I have read this submission and believe that I have an appropriate level of expertise to confirm that it is of an acceptable scientific standard.
